# A multiplexed targeted mass spectrometric assay for quantifying obesity- associated biomarkers in human plasma

**DOI:** 10.21203/rs.3.rs-9588456/v1

**Published:** 2026-05-20

**Authors:** Tai-Tu Lin, Panshak Dakup, Athena A. Schepmoes, Thomas L. Fillmore, Adam C. Swensen, Shane S Kelly, Tong Zhang, Jie Pu, Jon M. Jacobs, James P. DeLany, Bret H. Goodpaster, Tujin Shi, Jun Qu, Wei-Jun Qian

**Affiliations:** Pacific Northwest National Laboratory; Pacific Northwest National Laboratory; Pacific Northwest National Laboratory; Pacific Northwest National Laboratory; Pacific Northwest National Laboratory; Pacific Northwest National Laboratory; Pacific Northwest National Laboratory; University at Buffalo, State University of New York; Pacific Northwest National Laboratory; Advent Health; Advent Health; Pacific Northwest National Laboratory; University at Buffalo, State University of New York; Pacific Northwest National Laboratory

**Keywords:** Obesity, biomarkers, LC-MS, multiplexed assay, human plasma, weight loss

## Abstract

**Background:**

Obesity is a major global public health challenge that contributes to numerous comorbidities and increased mortality. A better understanding of the biological mechanisms and disease risks associated with obesity requires robust monitoring of key circulating biomarkers, including adipokines, apolipoproteins, and inflammatory proteins. Targeted mass spectrometry (MS) offers a promising platform for developing specific, standardized, and multiplexed assays for biomarker quantification. In this study, we developed a multiplex targeted MS assay for the quantification of 42 obesity-associated biomarker candidates in human plasma.

**Methods:**

The assay was optimized for surrogate peptide selection, digestion incubation time, and LC gradient, and evaluated for linearity, lower limit of quantification (LLOQ), imprecision, and stability. A semi-automated sample preparation workflow using 96-well plates was also established to support high-throughput implementation. The assay was applied to a clinical cohort undergoing weight loss interventions to evaluate differences in protein abundance across obesity-related groups and to monitor biomarker changes over time. Statistical analyses were performed to identify proteins with significantly different abundances between study groups and before versus after intervention.

**Results:**

The assay demonstrated acceptable linearity, LLOQ, imprecision, and stability. Inter-laboratory validation using samples from 70 healthy individuals showed strong correlation with the finalized standard operating procedure (SOP). Application of the assay to an obesity cohort revealed significant differences in the levels of 6 proteins across obese, overweight, and healthy control groups, as well as 16 proteins that were differentially abundant in obese individuals compared with non-obese individuals (overweight and healthy controls combined). In subjects undergoing weight loss interventions, four proteins—CRP, PRG4, SERPINF1, and SHBG—showed significant concentration changes in individuals who achieved > 5% weight loss.

**Conclusions:**

These results demonstrate the robustness and high-throughput capability of this multiplex targeted MS assay for measuring obesity-associated plasma biomarkers. The assay shows potential clinical utility for improving the diagnosis, stratification, and risk assessment of obesity-related conditions, as well as for monitoring responses to weight loss interventions.

## Background

The prevalence of obesity has increased dramatically in recent decades, reaching an epidemic proportion in both children and adults [1, 2]. Obesity is a significant risk factor in contributing to the onset of multiple diseases and their increased morbidity and mortality [3]. Diseases that have seen a parallel rise alongside the obesity epidemic trajectory include cardiovascular diseases (CVD), arthritis, type 2 diabetes (T2D), and certain types of cancers [4–6]. While individual biomarkers have been linked to these associations, the broader pathophysiological mechanisms connecting obesity to these diseases remain poorly understood. Consequently, we aim to measure a panel of circulatory biomarkers linked to obesity and its related diseases to better elucidate the underlying mechanisms and support improved monitoring of disease progression and prognosis [7]. Towards such goals, it is essential to develop reliable multiplexed assays capable of precisely quantifying a sizable and diverse panel of biomarkers—including hormones, adipokines, myokines, and inflammatory mediators—that are integral to both the pathology of obesity and its connection to various diseases.

Protein biomarkers in serum or plasma are commonly measured in clinical settings using immunoassays. For example, C-reactive protein (CRP) and transferrin (TF) are used to assess inflammation and iron overload, respectively [8, 9]. However, the major drawbacks of immunoassays is the requirement for highly specific antibodies, which are not always available for protein/proteoform of interest and may be prone to cross-reactivity and non-specific binding [10]. In contrast, liquid chromatography-mass spectrometry (LC-MS) combined with selected reaction monitoring (SRM) represents a promising platform for moderate- to high-throughput protein quantification, delivering both high specificity and robust multiplexing capability [11, 12]. LC-SRM relies on the selection of unique surrogate peptides of specific target proteins, enabling the simultaneous quantification of multiple biomarkers across a broad dynamic range in complex samples such as human plasma [13].

Recently, an LC-SRM assay was reported for quantifying 8 adipokines and 10 apolipoproteins as key regulators and potential biomarkers for obesity in human serum samples and cell culture supernatants of human adipocytes [14]. Adipokines and apolipoproteins are key mediators that regulate body weight, glucose homeostasis, and lipid metabolism, and their circulating levels are altered in obesity [15]. In addition, the accumulation of macronutrients in the obese adipose tissue stimulates the secretion of inflammatory mediators [16]. Hence, there is an opportunity to expand the monitoring of a landscape of regulatory players and obesity-associated biomarkers to provide deeper insights into biomarkers for obesity and associated diseases.

In this work, we aim to establish a multiplexed LC-SRM assay for precise monitoring of a relatively large panel of obesity biomarkers in circulation. The selection of the biomarker panel was mainly based on extensive literature mining and detectability in human plasma. The final panel contains adipokines, apolipoproteins, pro- and anti-inflammatory proteins, and glucose homeostasis regulator proteins. We demonstrated that the optimized assay provided reproducible and quantitative measurements of 42 obesity biomarkers, including validation of reproducibility through inter-laboratory comparisons. The clinical utility of the assay was further demonstrated using samples from an obesity study cohort.

## Methods

### Experimental Design and Rationale

**Figure 1:**
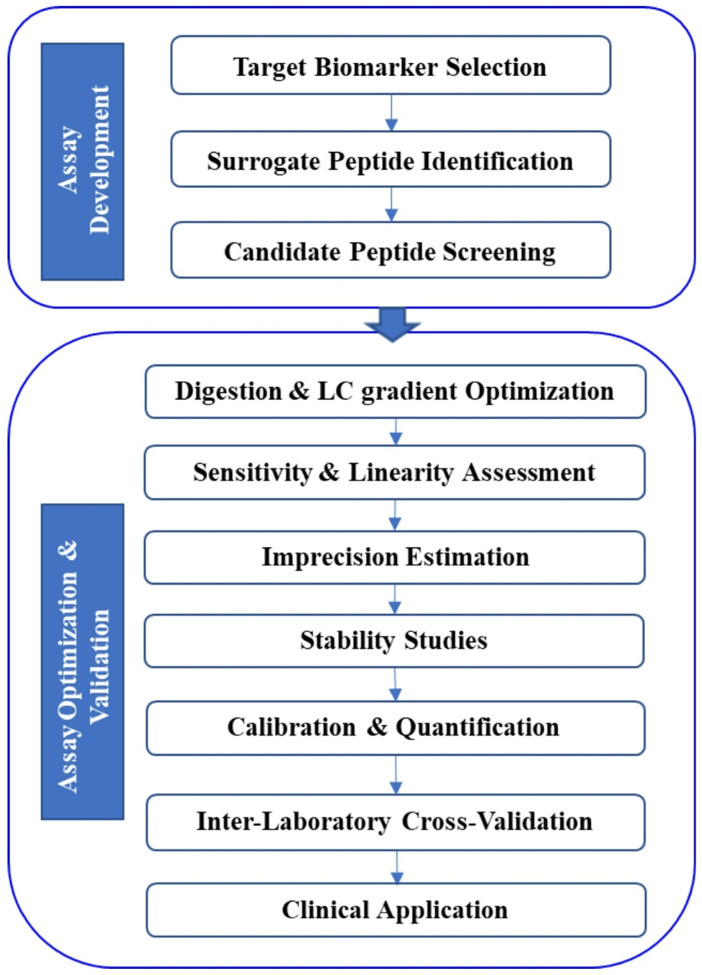
Strategy for developing a selective reaction monitoring (SRM)-based multiplex obesity biomarker assay.

The objectives of the study were to i) develop a direct LC-SRM-based multiplex assay for quantification of a panel of obesity-associated biomarkers in blood and ii) demonstrate the robustness and utility of this assay using a cohort of plasma samples from obesity patients undergoing clinical interventions. The overall workflow of assay development and validation is summarized in Fig. 1.

### Reagents and Samples

A commercially available pooled mixed-sex human plasma (K2EDTA anticoagulant) was purchased from BioIVT (Westbury, NY) and used as a reference plasma sample. Stable-isotope standard (SIS) peptides labeled with ^13^C/^15^N on C-terminal lysine or arginine for all targeted proteins were purchased from Biosynth (Gardner, MA). All cysteine residues in the SIS peptides were carbamidomethylated. Urea, dithiothreitol (DTT), iodoacetamide (IAA), ammonium bicarbonate, trifluoroacetic acid (TFA) and formic acid were obtained from Sigma (St. Louis, MO). Trypsin was obtained from Promega (Madison, WI).

De-identified plasma samples were from an obesity study cohort provided by the Translational Research Institute, Advent Health (Orlando, FL). Cross-sectional samples were from subjects with a wide range of body mass index (BMI) and longitudinal samples from a subset of subjects with obesity undergoing either a 12-month low-calorie diet plus exercise intervention or a 6-month low-calorie diet followed by a 6-month low-calorie diet plus exercise intervention (trial identifier: NCT00712127) were included for analysis. Analysis of the de-identified samples in this study was qualified as non-human subject research by the Institutional Review Board (IRB) at Pacific Northwest National Laboratory.

### Surrogate Peptides and Transitions Selection

A mixture of crude heavy peptides at 500 fmol/μL for each peptide was created by mixing stock solutions of the heavy peptides (2 nmol/μL in 15% ACN) and analyzed by LC-MS/MS using higher-energy collisional dissociation (HCD) on a Thermo Scientific Orbitrap Velos mass spectrometer coupled to nanoACQUITY UPLC system (Waters, Milford, MA) to check peptide quality and obtain information on peptide fragmentation (i.e., transitions). The Skyline software was used to build the peptide library [17]. The seven most intense fragment ions determined by HCD analysis of each candidate were selected for LC-SRM assay (**Supplemental Table 2**). The quality of heavy peptides was initially assessed based on their signal, peak shape, and stability in retention time. The final list of heavy peptide transitions was assessed using a scheduled LC-SRM method, with fewer than 500 transitions per event (**Supplemental Table 3; 10** min retention time window).

### Preparation of Plasma Samples

All human plasma samples were subjected to standard protein digestion workflow. External quality control (QC) was implemented using a reference sample of pooled plasma. Briefly, 5 μL of plasma sample was added to each well (or tube) containing 45 μL of 8 M urea in 50 mM NH_4_HCO_3_. Then, 5 μL of 100 mM DTT was added to each well and incubated for 30 mins at 37°C for sample reduction; followed by adding 5.5 μL of 400 mM IAA and incubating in the dark for 1 hour at 37°C for alkylation. The samples were diluted 6-fold by adding 50 mM NH_4_HCO_3_ containing 1 mM CaCl_2_. Trypsin was added to achieve an enzyme-to-substrate ratio of 1 μg of Trypsin per 50 μg of total protein and incubated for 6 hours at 37°C. The enzymatic digestion was finally quenched by acidifying each sample to a final TFA concentration of 0.1%. 50 μL of digested peptides of each sample were transferred to a new tube. The SIS mixture of 50 fmol/μL for each peptide was prepared, and 10 μL of the SIS mixture was added to each sample in the new tube. The tubes containing the samples and SIS peptides were stored at −70°C until SPE cleanup. The SPE cartridge was pre-washed with 1.5 mL of 100% MeOH and conditioned with 1.5 mL of 80% ACN, 0.1% TFA followed by 1 mL of 0.1% TFA. The digested peptide samples were loaded onto the SPE cartridge, washed with 2 mL of 0.1% TFA, and eluted into a 1.5 mL tube using 0.4 mL of 80% ACN, 0.1% TFA. The eluted peptides were dried in a speed-vac and stored at −80°C until further analysis.

For higher throughput sample processing, a 96-well plate-based digestion was employed to include 70 individual samples and 18 external QC samples randomized in wells across the plate, leaving positions A1 to H1 for BCA assay standards (**Supplemental Fig. 5**). We followed previously established protocol for digestion and cleanup outlined above [18]. After the digestion, 50 μL (~ 10% of total volume) of digested peptides of each sample were transferred to a new plate and 10 μL (50 fmol/μL) of the SIS mixture was added to each sample. For SPE clean-up, we employed the Strata c18-E SPE 96-well plate (Phenomenex, CA). The samples were eluted into a 0.5-mL Axygen plate using 0.4 mL of 80% ACN, 0.1% TFA. The eluted peptides in the plate were dried down and reconstituted using 100 μL Nanopure H_2_O for BCA assay. The sample was then stored at −80°C until SRM analysis.

### LC-SRM Analysis

The LC-SRM analysis was performed on a nanoACQUITY UPLC system (Waters Corporation, Milford, MA) interfaced to a TSQ Altis QQQ mass spectrometer equipped with a nano-ESI source (Thermo Scientific). An injection volume of 2 μL was used for the LC separation using a BEH C18 nanoACQUITY column (100μm id×100mm, 1.7μm, 130 Å, Waters). The mobile phase A was 0.1% FA in water and mobile phase B was 0.1% FA in ACN. The LC gradient included a linear gradient 1–6% B over 5 min with a flow rate of 500 nL/min followed by ramping 6–13% B in 5–20min, 13–22% B in 20–35 min, 22–40% B in 35–40 min, 40–95% B in 40–42 min, and maintaining 95% B in 42–47 min. The TSQ Altis was operated in positive ion-mode with the ESI voltage set to 2,100 V and a capillary inlet temperature of 350°C. Tube lens voltages were obtained from automatic tuning and calibration without further optimization. Both Q1 and Q3 were set at unit resolution of 0.7 FWHM, and Q2 gas pressure was 1.5 mTorr. The optimized LC-SRM method with precursor and production m/z values, CE, retention time was conducted scheduled SRM method with RT window set to be 15 min and cycle time of 1 s.

### Assay Validation

The analytical method validation experiments included evaluation of linearity, lower limit of quantification (LLOQ), imprecision, stability, and cross-lab comparison.

#### Calibration:

For quantification, an external nine-point calibration curve was generated. This was achieved by spiking a digested equine serum matrix with a dilution series of high-purity (> 99%) light synthetic peptides at concentrations of 0.02, 0.1, 0.5, 1, 2, 10, 20, 40, 200 fmol/μL. A fixed concentration (20fmol/μL) of their corresponding crude-purity (> 75%) isotope-labeled internal standards was included in each point. Following LC-SRM analysis, all calibration curves exhibited excellent linearity, with coefficients of determination (R^2^ > 0.99) between the measured light-to-heavy (L/H) peak area ratios and the known light peptide concentrations (Supplemental Fig. 6). These curves were subsequently used to calculate the absolute concentration of each target peptide from the L/H ratios measured in the analytical samples. In addition, protein-level calibration was carried out using three recombinant proteins: CRP, RBP4 and APOH. These recombinant proteins were spiked into equine serum then subjected to the standard sample preparation procedure as described in the previous section.

#### Linearity and lower limit of quantification (LLOQ):

Assay linearity was evaluated using an eight-point dilution series created by mixing human plasma with equine plasma to achieve final human plasma concentrations ranging from 0.2% to 100% (100%, 50%, 10%, 5%, 2%, 1%, 0.5%, and 0.2%). Each dilution point was prepared in triplicate. A fixed concentration of the heavy isotope-labeled peptide mixture (20 fmol/μL) was spiked into each sample prior to LC-SRM analysis. Linearity was assessed by performing a linear regression of the measured light-to-heavy peak area ratios against the theoretical plasma concentrations. The Pearson correlation coefficient (r) was calculated to confirm linearity across the dynamic range and response factor was calculated to account for differences in the mass spectrometer’s response across varying sample concentrations [19]. Acceptable response factors range from 0.8 to 1.2, indicating detector responses that are reasonably close to ideal.

#### Imprecision:

To estimate total variability, we processed five replicates of digested peptides from the reference plasma sample on each of 5 days. Data for imprecision are presented as the coefficient of variation expressed in percentage (%CV). The mean intra-assay (CV_intra_, 5 replicates measured in a single day) and inter-assay (CVinter, 5 replicates measured across 5 days). The total variability was determined as: CV_total_ = [(CV_intra_)^2^ + (CV_inter_)^2^]^1/2^.

#### Stability:

Peptide stability was assessed with pre- and post-sample processing under varying storage durations, conditions, and freeze-thaw cycles. For pre-sample processing, the reference plasma sample was aliquoted into 12 vials, of which 3 were stored at ~ 4°C. From these 3 vials, 5 μL of plasma sample was aliquoted from each and digested to peptides immediately (0 day), at 24 hours (1 day), and at 48 hours (2 days). The other 9 vials were stored in a freezer at − 80°C. At week 1, 6 vials were thawed at room temperature for one hour, 3 vials were digested, while the other 3 vials were refrozen at −80°C. At week 2, the refrozen vials from week 1 were thawed for one hour and digested after two freeze-thaw cycles. Finally, in week 4, the last 3 vials were thawed to and digested after their first freeze-thaw cycle. Assessment of stability from post-processing measurements also followed a similar workflow, the digested reference plasma sample was aliquoted into 12 vials. The first three aliquots were stored in an autosampler at 4°C and analyzed at three time points: immediately (0 hours), 24 hours, and 48 hours. The other 9 vials were stored in a freezer at − 80°C. At week 1, 6 vials were thawed at room temperature for one hour, 3 vials were analyzed after one freeze-thaw cycle, while the other 3 vials were refrozen at −80°C. At week 2, the refrozen vials from week 1 were thawed for one hour and analyzed after two freeze-thaw cycles. Finally, in week 4, the last 3 vials were thawed to and analyzed after their first freeze-thaw cycle. Data for stability is presented as %CV.

#### Interlaboratory-laboratory assessment:

Laboratory site 1 (PNNL) used a nanoLC-MS system as described above. Laboratory site 2 (University at Buffalo) utilized an UltiMate 3000 LC system (containing SRD-3400 degasser, NCS-3500RS CAP pumps and a high-flow binary gradient pump, and WPS-3000TBRS autosampler with a 250-uL loop) coupled to a TSQ Quantiva via an Ion Max NG ion source with H-ESI probe and 34-G narrow-bore spray needle in positive-ion mode. 40 μL of each sample was loaded onto a C8 column (15 × 2.1mm, 3.5-μm particle size, 100 Å, Agilent) at flow rate of 1 mL/min for 0.5 min at 96% mobile phase A (water : acetonitrile : formic acid of 98:2:0.1, v/v/v) and 4% mobile phase B (water : acetonitrile : formic acid of 15:85:0.1, v/v/v) using the high-flow binary pump as sample trapping. Then the peptides are reversely eluted from trapping column to analytical C18 column (150× 0.5 mm, 2.2 μm, 130 Å, customized packed) at a lower flow rate of 25 μL/min, with an LC gradient as described above. The separation temperature was controlled at 40°C. A ZDV 6-port valve placed in the heated column compartment was utilized to coordinate operation of the two flow systems. The spray voltage was set to 3.5 kV, the vaporizer temperature to 50°C, the sheath gas flow to 8.0 arbitrary units, the auxiliary gas flow to 6.0 arbitrary units, and the capillary temperature was maintained at 325°C.

### Data Analysis

The LC-MS raw data were analyzed using Skyline [17]. Peak detection and integration were based on the same retention time and relative peak intensity ratios across multiple transitions between light peptides and heavy peptide standards. All data were manually inspected to ensure correct peak detection and accurate integration. Light to heavy (L/H) peak area ratios were used to quantify the target peptides. The final endogenous peptide concentrations (μg/mL) for all the samples were calculated using the response curves (**Supplemental Fig. 6**). Linear regression, Pearson correlation, and grouped statistical comparisons were carried out using GraphPad Prism 10.1.0 and R 4.3.2 for statistical and ROC curve analysis. Disease classification performance of the 42-biomarker panel was evaluated using ROC-AUC from a penalized logistic regression machine learning model. Modeling was performed in R packages tidymodels and glmnet. Data were split into training (75%) and test (25%) sets stratified by diagnosis. Each biomarker value was z-score standardized, and class imbalance in the training set was addressed using SMOTE upsampling. The model was tuned using 10-fold cross validation with folds stratified by disease diagnosis. After hyperparameter specification, the final model was trained on the full training set and evaluated for ROC-AUC using the test set.

## Results

### SRM Assay Development and Characterization Obesity Biomarker Candidate Selection

Prior literature served as an important resource for the selection of obesity-associated biomarkers [20]. Herein we used the ‘Genescraper’ R package [21] to comprehensively mine the published literature for potentially relevant targets with the keywords ‘biomarker’ and ‘obesity’. A total of 1,437 biomarker candidates were found. We then scored and ranked these candidates by biomarker annotation [22, 23], detectability based on MS-plasma proteome databases [24–28], and biological meaning [29–35] (see workflow in **Supplemental Fig. 1**). In total, 73 obesity biomarker candidates were selected for assay development (**Supplemental Table 1**).

### Surrogate Peptide Selection

Proteolytic surrogate peptide selection for each candidate protein is a critical step towards establishing sensitive, selective, and reliable SRM assays. The selection was based on a combination of information from in-silico prediction by Peptide Prediction with Abundance (PPA) [36] and CONSeQuence (CONS) [37], public data repositories (PeptideAtlas) [38], and previous publications [24–28]. In general, the selection criteria for peptides included i) 7 to 25 amino acids in length, ii) uniquely represent the target proteins, iii) no missed cleavage, and iv) contained no known post-translational modifications (PTMs). The online repository, PeptideAtlas, was used to rank the peptides using Empirical Suitability Scores, derived from prediction probability and MS experimental observations [38], in addition to spectral counts from published plasma proteome databases [24–28]. Finally, three to seven candidate peptides for each target protein were selected and synthetic crude heavy peptides were purchased (**Supplemental Table 1**).

### LC-SRM Analysis of Target Peptides

To examine the detectability of the 73 obesity biomarker candidates, we used tryptic digests from a pooled plasma sample for the LC-SRM evaluation. In total, the initial LC-SRM assay contained 2,408 transitions from 402 peptides covering 73 proteins. LC-SRM analysis of naive plasma peptides (~ 0.5 μg) mixed with 402 SIS peptides (200 fmol/μL each) showed the detection of 42 endogenous candidate proteins (**Supplemental Table 2**). Our criterion for determining the detectability of each candidate protein in plasma requires that at least two distinct peptides from the same protein be detected. Furthermore, the ratio dot product (rdotp) between the transition peak areas of light and heavy must be > 0.9 for at least five transitions for each peptide (**Supplemental Fig. 2**). The final 42 candidate panel comprised 10 Apolipoproteins, 15 pro-inflammatory and 4 anti-inflammatory proteins, 8 glucose homeostasis & energy balance proteins, 4 blood regulators, and 1 hormone-binding protein (Table 1).

### Optimization of Protein Digestion and LC Gradient

To ensure optimized generation of peptides, a digestion time-course analysis was performed on the selected quantitative peptides. We observed an increase in SRM signals proportional to incubation time till 6 hours. There were no significant increases between 6 to 18 hours, with some peptides showing decreasing intensity with 18 h of digestion (**Supplemental Fig. 3**). Thus, 6 hours digestion of plasma samples was set as our standard procedure.

To enhance the throughput of the assay, we evaluated the performance of shorter (35- and 29-minute) scheduled SRM gradients against the original 72-minute method. Across triplicate analyses of pooled QC samples, the quantitative peptide (L/H) ratios obtained with the shorter gradients showed strong correlation with the results from the original 72-minute run. (**Supplemental Table 3**). As the LC gradient decreased, the signal-to-noise (S/N) ratio and the lower limit of quantification (LLOQ; estimated by percentage of endogenous dilution) improved as expected (**Supplemental Table 3** and **Supplemental Fig. 4A**). Notably, the CV of all 42 quantitative peptides across the tested LC gradients remained below 20% (**Supplemental Table 3** and **Supplemental Fig. 4B**). Based on these results, the 35-minute LC gradient was selected for this assay.

### Linearity and Accuracy

**Figure 2:**
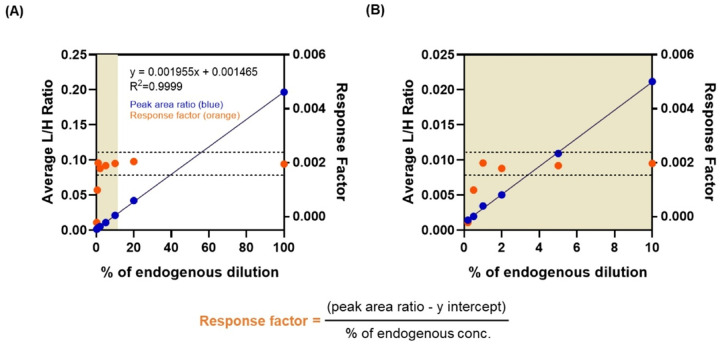
Linearity and Linearity and lower limit of quantification (LLOQ) determination using dilution experiments with equine plasma. (A) Representative linear response plot for the IGFBP3 peptide CQPSPDEARPLQALLDGR. Response factors remained within 20% of the analyte response (slope) down to 1% of the endogenous concentration. (B) Zoomed-in view of the 0.2%–10% endogenous concentration range.

To evaluate the linearity and lower limit of quantification (LLOQ) of endogenous analytes, we mixed the reference plasma sample with an analyte-free matrix of equine plasma and created an 8-point dilution series (100%, 20%, 10%, 5%, 2%, 1%, 0.5% and 0.2% of endogenous dilution). The top 3 transitions per peptide were used in quantification, the ratio of L/H. To avoid over-representing the degree of linearity in our dilution study, we combined linear regression analysis with the response factor plots to identify analyte concentrations that did not respond in a linear manner (Fig. 2). Peptides with a correlation coefficient of r > 0.99 and a maintained linear concentration response beyond 10X dilution were considered as quantifiable peptides with an acceptable linear dynamic range. Among 95 surrogate peptides for the 42 candidates, 86 peptides possessed good linearity (r^2^ > 0.99) and showed a LLOQ that was below 10X dilution of the reference sample. All the 42 candidates had at least one peptide with a good linear range (< 10% endogenous concentration) for further analytical assessments (**Supplemental Table 3**).

To further confirm the detection of the 42 biomarker candidates, individual plasma samples from 6 healthy subjects were used to monitor the 95 peptides. 88 peptides which were detected (ratio is higher than the LLOQ) in 5 of 6 individual plasma samples were selected as the surrogates for the candidates. From this test, all 42 candidates have at least one peptide that could be detected in all 6 individual plasma samples (**Supplemental Table 4**).

### Imprecision

To characterize the analytical precision of the LC-SRM measurements, we conducted a 5 X 5 imprecision study which we analyzed 5 replicates of plasma samples on each day for 5 days. Among the 88 peptides tested, 85 peptides displayed intra (within day), inter (across days) and total (combined) assay CV under 20% (**Supplemental Table 5**). As shown in Table 1, The final 42 peptides for final quantification all have %CV_total_ less than 20%.

### Stability

Next, we evaluated the stability of the assay measurements under different sample storage times and conditions. Reference plasma samples, along with digested reference plasma samples were aliquoted into 12 vials. For pre-extraction test assessing short-term stability, reference samples were prepared at three points: immediately (0 hours), after 24 hours, and after 48 hours. To evaluate long-term stability, reference samples were prepared at three intervals: one week, two weeks and four weeks incorporating 2 freeze-thaw cycles. For post-extraction test, digested reference plasma samples were subjected to a similar workflow. Short-term stability was assessed at time points of 0 hours, 24 hours, and 48 hours, while long-term stability was evaluated at one week, two weeks, and four weeks, including two freeze-thaw cycles. As shown in **Supplemental Table 6**, the CVs were calculated using the L/H ratio in three replicates under each sample storage conditions. In addition, all peptides were with CV under 20% CV across different sample storage conditions. It indicated the plasma samples and peptides were stable in 4°C for at least 48 hours and in − 80°C freezer for at least 4 weeks and through 2 freeze-thaw cycles (**Supplemental Table 6**).

### Semi-automated Workflow

To achieve a high-throughput and reproducible multiplexed assay, we evaluated the utility of a 96-well plate with automated digestion and SPE cleanup for sample preparation (**Supplemental Fig. 5**). A total of 70 individual plasma samples from healthy subjects and 18 technical replicates of the reference plasma were used for assessing the 95 peptides for 42 candidates. Measurements from 2 samples (2 individual plasma) were outside the normal distribution range as determined by an outlier test and were thus removed from the final assessment. **Supplemental Table 7** provides the quantification results for all 42 candidates across the 18 pooled replicates. For the 18 technical replicates, 92 of the 95 peptides displayed stable levels with CV ≤ 20%, suggesting good reproducibility of the entire workflow. As expected, the precision was associated with the SRM signal intensity, with the more intense ion signals showing less CV (**Supplemental Table 7**). For each protein target, we next selected the peptide with the lowest CV for further assay optimization (Table 1).

### Assay calibration

**Figure 3:**
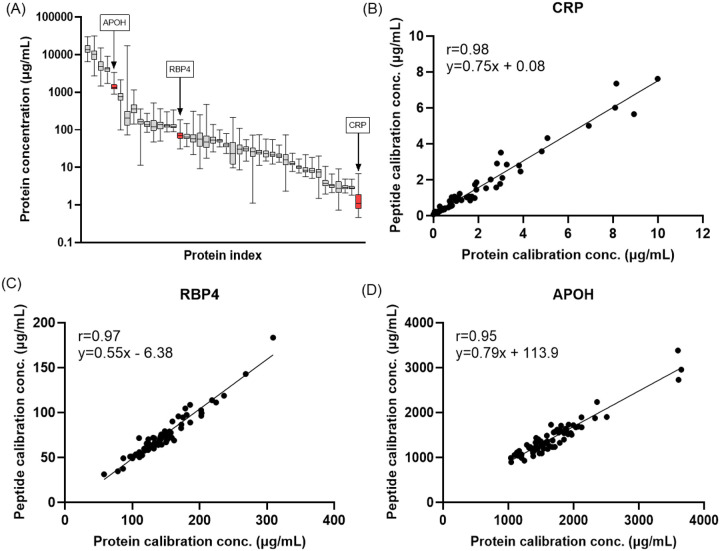
Protein quantification by peptide- and protein-based calibration. (A) Dynamic concentration range and inter-individual variability of target proteins estimated by peptide-based calibration. Concentrations from peptide-based calibration were compared by Pearson correlation (R) against concentrations from protein-based calibration in 3 proteins, CRP (B), RBP4 (C), and APOH (D) in 70 individual plasma samples.

To enable the LC-SRM assay for determining protein concentrations, we conducted calibration experiments and further quantified these 42 obesity biomarkers in the 70 individual plasma samples. After obtaining SRM L/H ratios from 70 samples, we used an external calibration curve generated using high purity (> 99%) light synthetic standard peptides (**Supplemental Fig. 6**) to allow the conversion of the measured L/H ratios in each sample to endogenous analyte concentration as μg/mL in plasma. Plasma concentrations for all quantified targets spanned across 5 orders of magnitude (from < 1 to > 10,000 μg/mL) and captured the inter-individual range of variability in expression in a representative healthy population (Fig. 3A). To further evaluate whether our peptide-level calibration would provide a close agreement with protein-level calibration, we used three purified recombinant proteins: CRP, RBP, and APOH, to obtain digested peptides which were analyzed by SRM to generate L/H ratios for protein concentration calculations. We compared concentrations determined by peptide calibration against measurements from recombinant proteins and found a strong positive Pearson correlation (r ≥ 0.95) for all 3 proteins (Fig. 3B–D). For RBP4, the concentration measured using the recombinant protein calibrant was approximately double that measured with the peptide calibrant, potentially due to differences in the purity of the recombinant proteins (not assessed). This suggests that our peptide-level quantification approach provides a close to accurate measurement of the quantity of our proteins of interest.

### Cross-lab validation of SRM assays for candidate biomarkers

**Figure 4:**
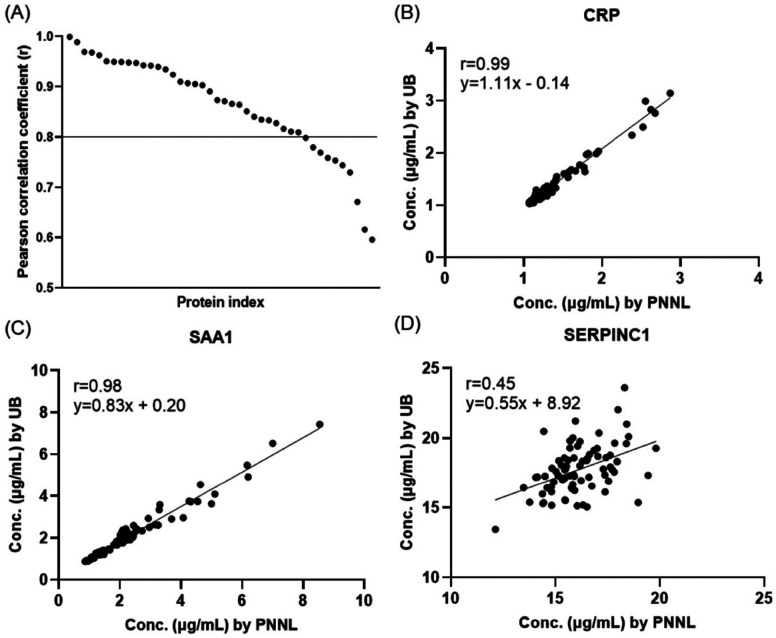
Inter-lab assay validation. The assay SOP was transferred and implemented at University at Buffalo (UB). The SRM assay results are presented for 252 transitions from 84 peptides of 42 proteins. For the 42 quantifiers, 32 of 42 proteins had strong correlation (r>0.8) while the remaining 10 had moderate correlation (0.6<r<0.8) between two inter-lab validation (A). The Pearson correlation coefficient was calculated in candidates, SAA1 (B), CRP (C) and SERPINC1 (D).

To further validate the reproducibility and robustness of our assay, we transferred and implemented the standard operation procedure (SOP) to a second laboratory site in the University of Buffalo, New York (UB). The same 70 individual plasma samples, reference plasma, and mixed SIS peptides aliquots were used for the comparison. The SOP was followed to process plasma samples and prepare the peptides for LC-SRM analysis, despite the use of different LC-MS platforms at the two laboratories. Out of the 42 proteins, 32 were found to have reproducible measurements and a strong positive Pearson correlation (r > 0.8; Fig. 4A.), even for low-abundant proteins such as SAA1 and CRP (Fig. 4B and 4C). However, the difference in separation platforms between the two sites (nano-flow LC at PNNL vs high-flow LC at UB) might have contributed to differences in the moderate positive correlation (0.6 < r < 0.8) for the remaining 10 proteins. In addition, the small dynamic range in concentration of certain proteins across individuals makes it more difficult to see a good correlation between two variables (Fig. 4D). Nevertheless, the inter-laboratory validation of our entire assay workflow to reproducibly measure ~ 80% of the targets with strong correlation demonstrates the overall robustness of the multiplex LC-SRM assay (**Supplemental Table 8**).

### Application of SRM assays to a Clinical Cohort of patient samples

**Figure 5:**
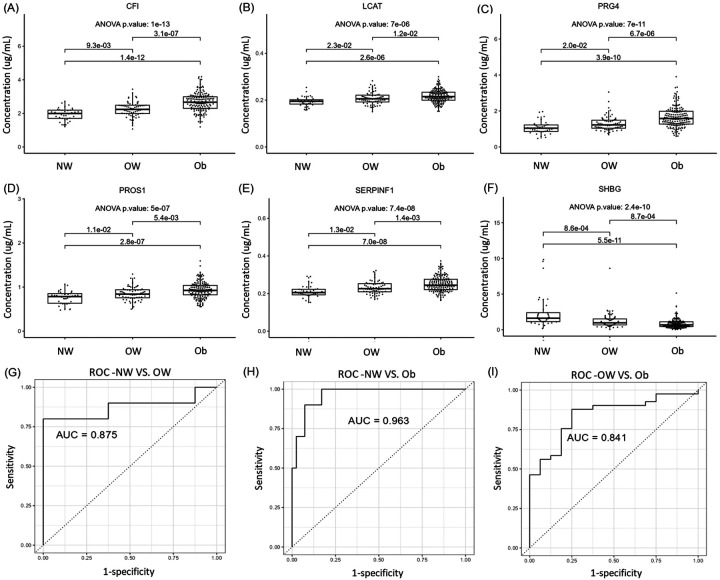
Application of the assay in a clinical cohort. Plasma samples from a clinical cohort study were processed and analyzed using the established 42-biomarker candidate assay. Proteins significantly differentiated across normal, overweight, and obesity groups, indicative of BMI association, included CFI (A), LCAT (B), PRG4 (C), PROS1 (D), SERPINF1 (E), and SHBG (F). Statistically significant group comparisons were determined using the one-way ANOVA test, with p-values represented by asterisks. The six-biomarker panel was further evaluated using ROC-AUC analysis, employing a penalized logistic regression machine learning model under conditions comparing NW and OW, NW and Ob, and OW and Ob. NW: normal weight; OW: overweight; Ob: obesity.

Next, we demonstrated the application of our assay in an obesity study cohort [39]. This cohort was comprised of three subpopulations based on the Body Mass Index (BMI) classification: a healthy control group termed ‘normal’ (n = 37) with a BMI of 18.5 to < 25, an ‘overweigh’ group (n = 63) with a BMI of 25 to < 30, and an ‘obesity’ group (n = 163) with a BMI > 30 (**Supplemental Table 9)** [40]. SRM analysis of plasma samples from these subjects revealed that concentrations of 6 out of 42 proteins were significantly higher (CFI, LCAT, PRG4, PROS1, and SERPINF1) or lower (SHBG) across all 3 groups in association with BMI (Fig. 5). These significant changes in protein concentrations were observed to follow a consistent increase or decrease in trajectory relative to the control group. Additionally, Receiver Operating Characteristic (ROC) showed that the 6 biomarkers could effectively distinguish between normal and overweight (AUC = 0.875), normal and obesity (AUC = 0.963), and overweight and obesity (AUC = 0.841). Another 16 proteins showed significant higher (AHSG, APOE, AOPH, ATRN, C1Q10, CP, CRP, F2, F10, FGB, HP, LBP, SAA1, and SERPINA3), or lower (APOD and APOM) in the obesity group relative to the overweight or control groups (**Supplemental Fig. 7**). Two proteins exhibited significant changes only in the obesity group relative to the overweight group (**Supplemental Fig. 8**). These findings provide a valuable metric to assess obesity biomarker candidates in the context of potential early/late-stage obesity coupled with BMI measurements.

**Figure 6:**
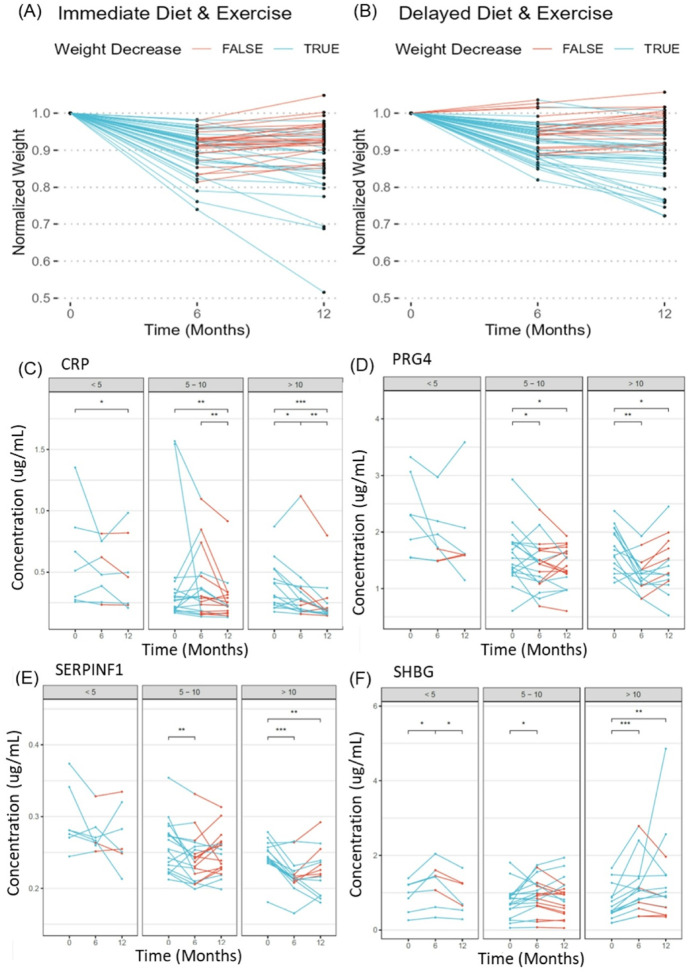
Assay application of the obesity 1-year longitudinal clinical plasma samples. The obesity subjects were placed under an immediate (A) or delayed (B) diet and exercise weight loss intervention. Plasma collected at baseline, 6 months, and 12 months were processed and analyzed for the established 42 biomarker candidate assay. Significantly reduced levels of CRP (A), PRG4 (B), and SERPINF1 (C) were observed in the group of >10% and 5–10% weight losses. The candidates SHBG (D) was significantly increased in the group of >10%, 5–10%, and < 5% weight losses. Statistically significant differences of group comparisons using One-way ANOVA test are shown as p-value asterisks.

From the same obesity cohort study, 80 patients (**Supplemental Table 10**) with obesity (BMI ≥ 30 kg/m^2^) who had no history of diabetes or cardiac-related diseases underwent either a 12-month low-calorie diet and exercise intervention (immediate diet and exercise) or a 6-month low-calorie diet followed by a 12-month low-calorie diet and exercise intervention (delayed diet and exercise). There were no observable differences in weight changes between the immediate and delayed diet and exercise interventions across 12 months (Fig. 6A–B). Longitudinal plasma samples collected at baseline, 6 months, and 12 months were used for our assay measurements. We stratified the subjects into three subgroups based on the extent of weight losses, independent of diet and exercise intervention timelines: those with > 10%, 5–10%, and < 5% weight losses. As shown in Fig. 6C–E, significantly reduced levels of CRP, PRG4, and SERPINF1 were observed in the subgroups of > 10% and 5–10% weight losses. SHBG was significantly increased in the subgroups of > 10%, 5–10%, and < 5% weight losses. Interestingly, these four markers displayed the most significant differences following the initial 6 months intervention of > 10% and 5–10% weight loss as opposed to the continual weight maintenance timeframe. A caveat in this comparison is the relatively small numbers of subjects in each category, which may not provide enough statistical power even if the analyte indeed responds to intervention. Overall, the data suggests that CRP, PRG4, SERPINF1, and SHBG are robust markers of obesity with substantial responses to weight losses, which also demonstrated the utility of the developed multiplex assay in clinical cohorts.

## Discussion

Although immunoassays are the most widely used platforms for clinical diagnostics, their utility in comprehensive biomarker research is hampered by significant inherent limitations. These include a lack of concordance across platforms, interference from autoantibodies and anti-reagent antibodies, and the high-dose hook effect, batch-to-batch variability, and limited multiplexing capacity [10, 41]. Several recent reports have highlighted failures of commonly used immunoassays to detect their intended targets, leading to false conclusions [42–44]. In contrast, SRM-based targeted proteomics offers a promising alternative for developing specific and standardized multiplex assays that do not rely on affinity reagents, by directly measuring surrogate peptides of the proteins of interest [12, 45]. This approach provides high specificity and enables the simultaneous measurement of numerous proteins and their proteoforms, making it ideal for profiling complex biomarker networks, such as those in obesity pathophysiology. Therefore, while immunoassays remain practical for routine, single-analyte clinical testing, SRM is superior for the rigorous, multiplexed biomarker discovery and validation required for advanced research.

In this study, we developed and validated an SRM-based targeted proteomics assay for the clinical evaluation of obesity-related biomarker candidates. Leveraging direct LC-SRM, the multiplexed assay is highly specific, accurate, reproducible, and does not require any affinity reagents. The LLOQ of the assay, ranging from 0.1 μg/mL to 87 μg/mL, is significantly below the reported endogenous interval ranges for these 42 biomarkers in human plasma. The measured concentrations were calibrated on the peptide level and confirmed with recombinant protein calibrants. Thus, this assay is sufficiently sensitive to reliably quantify these biomarker candidates. Furthermore, the method has been demonstrated to be transferable and can be easily standardized across laboratories. In the inter-laboratory validation study, two different MS laboratories using the same SOPs achieved strong positive correlation in 32 of 42 biomarkers, with the remaining 10 at moderate positive correlation.

In applying our assay to a cohort of 263 BMI-matched individuals, we find distinctive patterns of plasma protein alterations across the different stages of BMI classifications and post-intervention weight loss categories. The proteins CFI, LCAT, PRG4, PROS1, and SERPINF1 showed a progressive increase from healthy controls to overweight and obesity groups, while SHBG progressively decreased. These trends strongly suggest the involvement of metabolic dysregulation, inflammation, lipid metabolism, and coagulation pathways. For instance, CFI is a critical regulator of complement activation and LCAT is a crucial enzyme in lipid metabolism and transport. Abnormalities to these key regulatory proteins within their respective pathways can contribute to chronic inflammation and dyslipidemia, which are characteristic of obesity conditions [46, 47]. PRG4 and PROS1 are associated with anti-inflammatory and anticoagulant roles, while SERPINF1 acts as an anti-angiogenic factor, and their dysregulation might reflect heightened responses to the chronic inflammatory environment observed in obesity progression [48–50]. Conversely, SHBG which plays a critical role in regulating the bioavailability of sex hormones, had an inverse relationship to BMI, consistent with prior studies [51, 52]. Elevated insulin levels suppress hepatic SHBG production, disrupting hormonal balance and contributing to the metabolic dysfunction often associated with increased BMI [53]. This further underscore the potential implications of sex-specific differences in understanding the pathophysiological mechanisms of obesity. In the group with obesity specifically, relative to healthy controls or overweight, the significant elevated levels of AHSG, APOE, APOH, ATRN, C1QA, CP, CRP, F2, F10, FGB, HP, LBP, SAA1, and SERPINA3 reflect enhanced systemic inflammation, acute-phase responses, immune activation, and coagulopathy commonly observed in obesity pathophysiology [7, 54]. For example, CRP, a widely recognized marker of inflammation, is often elevated in obesity, underscoring the persistent inflammation that drives metabolic complications such as insulin resistance and cardiovascular disease [55]. Meanwhile, reduced levels of APOD and APOM demonstrate perturbations in lipid metabolism and transport pathways [56, 57].

Longitudinal data from within the cohort with obesity further reveal dynamic plasma protein adaptations in response to weight loss interventions. Weight reductions of > 10% and 5–10% resulted in significant decreases in markers such as CRP, PRG4, and SERPINF1. This potentially reflects diminished inflammation and improved metabolic regulation with substantial weight loss. Notably, SHBG levels increased across all weight loss groups. For all these proteins, the largest changes were observed during the initial six-month intervention phase. These changes during the first six months suggest that the effects of weight loss strategies are more pronounced during active intervention phases compared to weight maintenance phases. Reduced CRP levels underscore the attenuation of systemic inflammation, changes in PRG4 and SERPINF1 indicate shifts away from pathological processes such as coagulation and fibrosis, while an increase in SHBG points to an improvement in hormonal balance and metabolic function. Our findings align with previous discovery approaches that identified proteins altered following weight loss interventions. For example, one study reported a significant decrease in CRP levels (−35%), SERPINF1 levels (−16%), and PRG4 levels (−19%), alongside an increase in SHBG levels (+ 117%) after a 12% reduction in body mass in a cohort of 43 obese individuals who underwent an 8-week weight loss intervention followed by one year of weight maintenance [58]. Another study demonstrated a strong association between CRP and BMI in a cohort of 1,002 overweight and obese individuals following an 8-week weight loss and a subsequent 6-month weight maintenance period [59]. Both studies utilized low-calorie diet protocols. In comparison, our cohort utilized a combined low-calorie diet and exercise intervention, which offers distinct advantages over diet alone. Exercise helps preserve lean muscle mass during weight loss, enhances energy expenditure, and improves insulin sensitivity, amplifying the overall metabolic benefits of a low-calorie diet [60]. Together, these strategies create a synergistic effect on reducing adiposity and mitigating obesity-associated inflammation and metabolic dysregulation. Importantly, our multiplexed assay provides robust validation for several biomarkers, including CRP, SERPINF1, PRG4, and SHBG, emphasizing their translational potential and clinical utility for monitoring therapeutic interventions in obesity and weight loss.

## Supplementary Files

Table 1 is available in the Supplementary Files section.

This is a list of supplementary files associated with this preprint. Click to download.

- MultiplexAssayofObesityBiomarkersSupplementalTables.xlsx
- SupplementalMaterialFinal.docx
- Table1.docx

## Data Availability

Associated Skyline files have been in Panorama under the TaMADOR-PNNL Group Site, and are accessible via: https://panoramaweb.org/cDPDns.url

## Abbreviations

BMI: Body mass index
CVD: Cardiovascular diseases
CONS: CONSeQuence
CRP: C-reactive protein
DTT: Dithiothreitol
IAA: Iodoacetamide
IRB: Institutional Review Board
L/H: Light-to-heavy
LOD: Limits of detection
LLOQ: Lower limit of quantification
PPA: Peptide Prediction with Abundance
QC: Quality control
ROC: Receiver Operating Characteristic
SRM: Selected reaction monitoring
S/N: signal-to-noise
SIS: Stable-isotope standard
SOP: Standard operating procedure
TF: Transferrin
TFA: Trifluoroacetic acid
T2D: Type 2 diabetes
